# Xyloglucan endotransglucosylase/hydrolases (XTHs) are inactivated by binding to glass and cellulosic surfaces, and released in active form by a heat-stable polymer from cauliflower florets

**DOI:** 10.1016/j.jplph.2017.07.022

**Published:** 2017-11

**Authors:** Sandra C. Sharples, Tu C. Nguyen-Phan, Stephen C. Fry

**Affiliations:** The Edinburgh Cell Wall Group, Institute of Molecular Plant Sciences, The University of Edinburgh, Daniel Rutherford Building, The King’s Buildings, Max Born Crescent, Edinburgh EH9 3BF, UK

**Keywords:** BCP, boiled cauliflower preparation (as defined by Takeda and Fry 2004), CHP, cold water-extractable heat-stable polymer, MES, morpholinoethanesulphonic acid, PL, polylysine, XAF, XTH activating factor, XET, xyloglucan endotransglucosylase (activity), XTH, xyloglucan endotransglucosylase/hydrolase (protein), XXXGol, NaBH_4_-reduced heptasaccharide of xyloglucan (xylose_3_·glucose_3_·glucitol), Xyloglucan, Cell wall modification, Arabinogalactan-proteins, Cellulose binding, Xyloglucan endotransglucosylase/hydrolase, Cell expansion

## Abstract

Xyloglucan endotransglucosylase (XET) activity, which cuts and re-joins hemicellulose chains in the plant cell wall, contributing to wall assembly and growth regulation, is the major activity of XTH proteins. During purification, XTHs often lose XET activity which, however, is restored by treatment with certain cold-water-extractable, heat-stable polymers (CHPs), *e.g.* from cauliflower florets. It was not known whether the XTH-activating factor (XAF) present in CHPs works by promoting (*e.g.* allosterically) XET activity or by re-solubilising sequestered XTH proteins. We now show that XTHs in dilute solution bind to diverse surfaces (*e.g.* glass and cellulose), and that CHPs can re-solubilise the bound enzyme, re-activating it. Cell walls prepared from cauliflower florets, mung bean shoots and *Arabidopsis* cell-suspension cultures each contained endogenous, tightly bound, inactive XTHs, which were likewise rapidly solubilised (within 0.5 h) and thus activated by cauliflower XAF. We present a convenient quantitative assay for XAF acting on the native sequestered XTHs of *Arabidopsis* cell walls; using this assay, we show that CHPs from all plants tested possess XAF activity. The XAF activity of diverse CHPs does not correlate with their conductivity, showing that this activity is not a simple ionic effect. The XAF action of cauliflower CHPs was augmented by NaCl, although NaCl alone was much less effective than a CHP solution of similar conductivity, confirming that the cauliflower polymers did not simply exert a salt effect. We suggest that XAF is an endogenous regulator of XET action, modulating cell-wall loosening and/or assembly *in vivo*.

## 1 Introduction

In dicot primary cell walls, the major tension-bearing structure, limiting cell expansion, is widely proposed to include a xyloglucan–cellulose complex (Fry, 1989, Hayashi, 1989, McCann et al., 1990, Passioura and Fry, 1992, Fenwick et al., 1999, Park and Cosgrove, 2015). Enzymes of xyloglucan metabolism are therefore of interest to our understanding of the control of wall strength and extensibility. In particular, the transient cleavage and rearrangement of xyloglucan chains is achieved by GH16-family enzymes named xyloglucan endotransglucosylase/hydrolases (XTHs), which are ubiquitous in land plants. The major activity of most XTHs is xyloglucan endotransglucosylase (XET; EC 2.4.1.207; Baydoun and Fry, 1989, Smith and Fry, 1991, Farkaš et al., 1992, Fry et al., 1992, Nishitani and Tominaga, 1992), though a few exhibit predominantly xyloglucan *endo*-hydrolase activity (XEH; Shi et al., 2015). The XET reaction involves the covalent ‘cutting and pasting’ of xyloglucan chains, which has been shown by *in-vivo* density labelling to occur during — and probably contribute to the mechanism of — cell-wall assembly (Thompson et al., 1997) and loosening (Thompson and Fry, 2001). Roles for XET activity in growth control, both stimulatory and inhibitory, have been demonstrated multiple times (Osato et al., 2006, Miedes et al., 2013, Maris et al., 2009, Liu et al., 2007, Van Sandt et al., 2007).

Although there have been many studies of XTHs and their enzymic activities, little is known about how XTH action is regulated *in vivo*, which may be crucial for plant cell growth control. The ionic environment of the enzyme within the apoplast may be one factor controlling the action of XTHs (Taleisnik et al., 2009, Han et al., 2013) and other wall enzymes (Goldberg et al., 1992, Almeida and Huber, 2007). Takeda and Fry (2004) reported the presence of an endogenous polymeric factor, extractable from cauliflower florets, that promotes the XET activity of a crude, de-salted cauliflower XTH preparation as well as heterologously produced XTH24. In the present paper, we refer to this factor as XAF (=XTH activating factor). The polymer was solubilised from the florets in cold water, and — unlike most proteins — it remained soluble after boiling; the preparation was therefore termed ‘boiled cauliflower preparation’ (BCP; Takeda and Fry, 2004). Although the active principle responsible for XAF activity was not identified, BCP preparations containing it were rich in arabinogalactan-proteins. In agreement with this, gum arabic (a crude arabinogalactan-protein preparation) exerted some XAF activity (Takeda and Fry, 2004). In contrast, cauliflower pectin and hemicellulose preparations had little effect. Therefore it was suspected that XAF was attributable to the arabinogalactan-protein content of BCP. Many inorganic and organic salts exhibited XAF activity, and the order of effectiveness of metal chlorides was trivalent > divalent > monovalent, suggesting an effect due to ionic strength. In addition, some but not all anionic polysaccharides exhibited high XAF activity — *e.g.* carboxymethylcellulose, pectin, gum arabic and hypochlorite-oxidised (thus anionic) xyloglucan, but not alginate, λ-carrageenan, homogalacturonan and methylglucuronoxylan (Takeda and Fry, 2004, Takeda et al., 2008). Thus, XAF activity was not simply a non-specific effect of any anionic polymers.

Takeda and Fry (2004) defined BCP as the total cold-water-extractable, heat-stable preparation from cauliflower florets. BCP, thus defined, contains numerous low-M_r_ substances including both inorganics (K^+^, Ca^2+^, phosphate etc.) and organics (sugars, citrate, amino acids etc.), as well as the relatively small amount of cold-water-extractable polymers that remained soluble on heating (≈8% of the total BCP dry weight). About half the XAF activity present in BCP was attributable to these polymers, and their effect was much higher than would have been predicted from their ionic strength (assayed by conductivity) in comparison with inorganic salts. Thus, the BCP polymers exerted a high XAF activity that was not due simply to their ionic strength. In the present work, we have used only the polymeric fraction, and ‘BCP polymers’ in the terminology of Takeda and Fry (2004) are referred to here as CHPs (cold-water-extractable, heat-stable polymers).

Takeda and Fry (2004) did not determine whether the ability of the XAF, present in CHP, to restore lost XET activity was due to a promotion (*e.g.* allosteric) of the activity of the enzyme or to a re-solubilisation of enzyme that had been sequestered in some way. As a step towards characterising XAF, we have now distinguished between these possibilities. We investigated the ability of XTHs to bind to various artificial and natural surfaces — including cellulose and native cell walls — and the ability of CHP and/or NaCl to re-solubilise (and thereby re-activate) bound enzyme. In this manuscript we also present a convenient new assay for XAF activity on *Arabidopsis* cell walls that contain native bound XTHs.

## 2 Materials and methods

### 2.1 Materials

Heterologously expressed AtXTH24, produced in baculovirus-infected insect cells in Sf-900 II serum-free medium (Invitrogen, Carlsbad, California) as described by Campbell and Braam (1999), was kindly supplied by Dr Janet Braam (Rice University, TX, USA). The total protein concentration in the collected medium was 325 μg ml^−1^, as estimated by the Bradford micro-assay (Bradford, 1976). *Tamarind* xyloglucan was a generous gift of Mr K. Yamatoya, Dainippon Pharmaceutical Co., Osaka, Japan. XXXGol was bought from Megazyme. [^3^H]XXXGol was from EDIPOS (http://fry.bio.ed.ac.uk/edipos.html) and when used carrier-free had specific radioactivity approximately 100 MBq μmol^−1^. It was routinely used as 0.5 or 1 kBq per assay. Other general chemicals were bought from Sigma.

### 2.2 Preparation of CHP

Cauliflower florets from a supermarket were vigorously homogenised in a blender (300 g in approximately 100 ml de-ionised H_2_O). The homogenate was filtered through three layers of Miracloth and the filtrate was incubated at 100 °C for 1 h then filtered through Miracloth again. The filtrate (crude extract) was frozen, thawed, mixed well, and centrifuged at 4000 rpm for 30 min, then the clear supernatant was mixed with 2.3 vol of 96% ethanol. After a second centrifugation, the supernatant was discarded and the pellet was further washed with, sequentially, 80% and 96% (v/v) ethanol. The pellet was air-dried (yield: 1.8–3.0 mg dry polymer per g fresh weight cauliflower), re-dissolved in a minimum volume of de-ionised water, and freeze-dried. The dried pellet was re-dissolved in water or 0.2 M MES (Na^+^), pH 5.5, at a concentration of 2 mg/ml (total dry weight of pellet per ml buffer), labelled CHP, and stored at −20 °C until use.

An identical procedure was applied to a selection of other plant materials (listed in Table 1). In the case of cell-suspension cultures, the whole culture (200 ml; 7–9 days old) was homogenised in the blender with no additional water. For determination of ionic strengths, dried CHPs were re-dissolved at 2 mg/ml in pure water and the conductivity was read with a Jenway 4060 conductivity meter.

**Table 1:** XTH-activating factor (XAF) activity of CHP preparations from diverse plant sources.

Source of CHP	Yield of CHP extracted (mg/g fresh weight)	XAF activity of 2 mg/ml CHP (cpm [^3^H]polysaccharide produced)^a^	Conductivity (mM NaCl equiv) of a 2 mg/ml CHP solution^b^
Watercress shoots	0.88	2980 ± 214	7.29
Parsley leaves	1.57	2587 ± 9	6.14
Arabidopsis leaves	nd	2582 ± 114	6.92
Tobacco leaves	2.40	2366 ± 190	4.88
Snowdrop leaves	0.64	2125 ± 82	5.35
Asparagus shoots	1.18	1710 ± 102	2.13
Tobacco stems	0.84	1640 ± 22	13.7
Crocus leaves	0.68	1573 ± 84	3.16
Arabidopsis stems	nd	1562 ± 40	6.40
Lettuce leaves	0.49	1528 ± 168	2.74
Snowdrop stems	0.36	1415 ± 54	4.96
Snowdrop flowers	0.68	1329 ± 12	3.13
Arabidopsis flowers + seeds	nd	1267 ± 126	5.55
Spinach leaves	2.41	1259 ± 8	15.5
Celery whole leaves (very young)	1.00	1054 ± 86	2.57
Spinach cell-culture	0.54	1036 ± 83	1.41
Carrot leaves	1.86	971 ± 86	6.97
Arabidopsis cell-culture	0.59	882 ± 18	2.39
Celery petioles (mature)	0.56	879 ± 39	1.92
Cauliflower	nd	872 ± 89	3.87
Spring onion basal leaves + stem	0.75	786 ± 171	2.35
Rose cell-culture	0.24	770 ± 30	1.70
Crocus flowers	0.67	614 ± 38	2.33
Spring onion young leaves	0.24	536 ± 44	4.98
Carrot roots	1.13	492 ± 41	1.65
Control (200 mM MES buffer only)	na	37 ± 4	(≤40.0)
Control (water)	na	36 ± 6	(<0.01)

nd, not determined; na, not applicable.

a Washed *Arabidopsis* cell walls (15 μg dry weight) were incubated in the CHP solution (66 μl; 2 mg/ml in 200 mM MES buffer, pH 5.5) from the indicated botanical sources for 30 min, then the solubilised wall enzymes (20 μl of the solution) were assayed for XET activity with 1 kBq [^3^H]XXXGol for 16 h in the presence of 2.5 mg/ml bovine serum albumin. The yield of [^3^H]polysaccharide is reported (two replicate assays; the error values indicate the range).

b CHP in unbuffered water. For comparison, note that 2 mg/ml pure NaCl is 34 mM.

### 2.3 Preparation of *Arabidopsis* cell walls containing native bound XTHs

*Arabidopsis thaliana* cell-suspension cultures were grown in the medium of May and Leaver, (1993) with 2% (w/v) glucose in place of sucrose. Cells were grown under continuous low-intensity fluorescent lighting (about 25 μmol m^−2^ s^−1^) in 500-ml conical flasks with constant shaking at 150 rpm. Sub-culturing was performed weekly by transfer of approximately 20 ml of old culture into 180 ml of fresh medium. A 200-ml portion of 7–9-d culture was homogenised for 2 min with a hand-held food mixer, then passed through Miracloth. The cells on the Miracloth were washed with 1 l of ice-cold water, squeezed semi-dry and frozen overnight at −20 °C. The cells were thawed and re-washed on Miracloth with another 1 l of ice-cold water. The cells were squeezed again and re-suspended in 100 ml de-ionised water. While the cell suspension was kept stirring, 1.5-ml aliquots were made and the aliquots were stored frozen at −20 °C for further use. Representative aliquots were freeze-dried for dry weight determination.

### 2.4 Radiochemical XET assay

Unless otherwise stated, the enzyme (in solution and/or bound to a glass or other surface) was supplied with a reaction mixture to give final concentrations of: 80–500 kBq/ml [^3^H]XXXGol, 100 μM non-radioactive ‘carrier’ XXXGol (thus final specific radioactivity of acceptor substrate = 0.8–5.0 MBq/μmol), 0.3% tamarind xyloglucan, 0.25% w/v chlorobutanol and 100 mM MES (Na^+^, pH 5.5). In some experiments, NaCl or other agents were also present. CHP, when present, was routinely at a final concentration of about 1.5 mg/ml. After incubation at room temperature for various times (1–24 h, depending on the aims of the experiment), a 20-μl aliquot was added to 20 μl 45% formic acid and dried on a 4 × 4 cm square of Whatman no. 3 paper (marked in pencil on a large sheet), which was then washed overnight in running tap-water and re-dried. The ^3^H-labelled polysaccharide product, remaining on the paper, was assayed by liquid scintillation counting (with 2 ml OptiScint scintillant per paper square; counting efficiency approx. 28%).

### 2.5 Simplified assay of XAF action on washed *Arabidopsis* cells

A suspension of water-washed *Arabidopsis* cell walls was dispensed into a 96-well plate, each well receiving 66 μl containing 15–18 μg (dry weight) of cell walls. The plate was centrifuged at 4000 rpm for 10 min and the supernatant was carefully discarded by use of tissue paper. The pellet in each well was then re-suspended in 66 μl of a putative XAF solution [routinely buffered with 0.2 M MES (Na^+^), pH 5.5]. After 30 min on a shaker at room temperature, the plate was again centrifuged and 20 μl of supernatant was transferred into a well in a new 96-well plate, where any solubilised enzyme was radiochemically assayed for XET activity as above.

## 3 Results

### 3.1 XTH24 is inactivated by binding to surfaces; CHP reactivates it by solubilisation

CHP and inorganic salts each exert ‘XAF’ activity — *i.e.* they increase the measured XET activity of a de-salted XTH preparation (Takeda and Fry, 2004). They could potentially achieve this by serving as a cofactor or allosteric activator of XTHs; alternatively they might prevent the enzyme’s denaturation or sequestration on the surfaces of assay vials. It has indeed often been noted that XTHs tend to adsorb to surfaces, including chromatography columns, reducing their recovery (Hrmova et al., 2007). In the following experiments we therefore set out to distinguish between these possibilities by studying the behaviour of an arbitrarily chosen XTH, AtXTH24 XTH, heterologously expressed in insect cells. XTH24 is not expected to be unusual among the 33 XTHs of *Arabidopsis thaliana*; it is highly expressed in young, growing tissues, *e.g.* hypocotyls (Campbell and Braam, 1999, Jamet et al., 2009) and floral parts especially the stamens (http://bar.utoronto.ca/efp/cgi-bin/efpWeb.cgi).

**Fig. 1:**
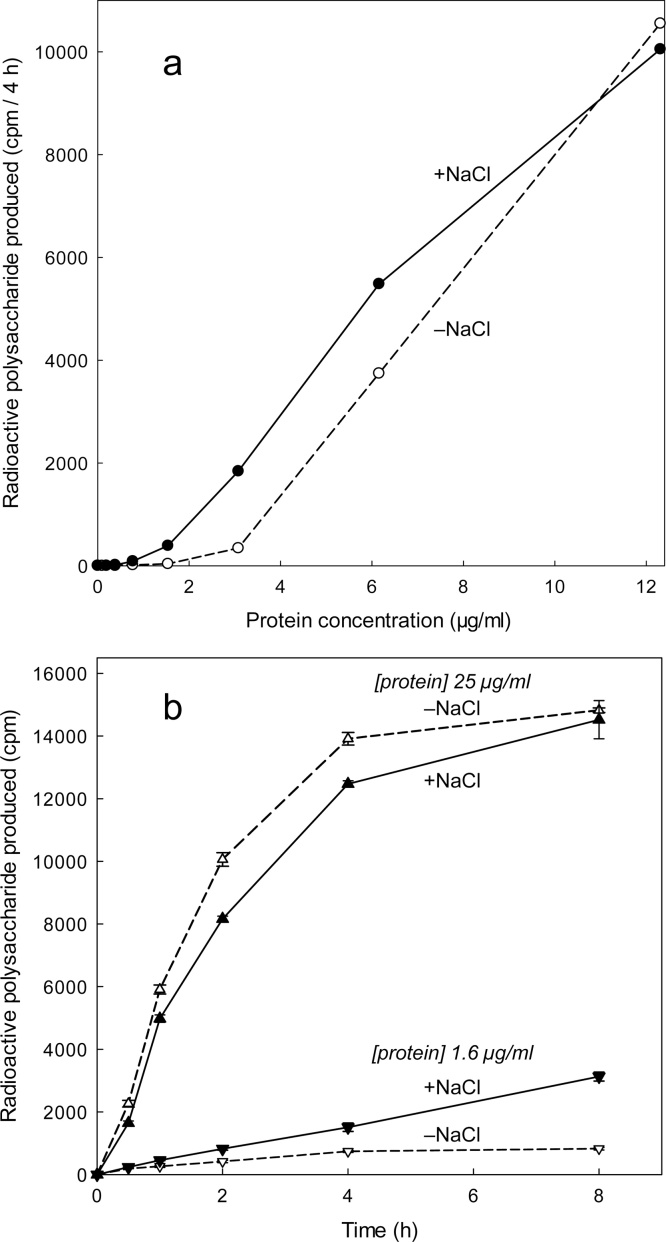
Non-linear relationship between XTH24 concentration and measurable XET activity. (a) The stock AtXTH24 solution was serially diluted in water (in polypropylene Eppendorf vials). The enzyme solution was then assayed for XET activity, in the same vials, in the presence and absence of 240 mM NaCl. (b) Time-course of XET action exhibited by two dilutions of stock XTH24 (protein concentration 2.1 and 25 μg/ml) in the presence and absence of 240 mM NaCl. This experiment was conducted twice, with similar results; one run is shown. In these experiments, the volume sampled had contained 1.0 kBq [^3^H]XXXGol (carrier-free); the theoretical maximum yield of polysaccharide product was about 17,000 cpm.

Fig. 1a shows a markedly non-linear relationship between the calculated AtXTH24 concentration and the measured XET activity. In the range 2.0–0.5% (v/v) of a stock XTH24 solution, there was a *ca*. 10-fold decrease in activity for every halving of concentration; there was very little measureable XET activity below a threshold concentration of ∼1% (v/v). The pronounced loss of activity observed at low concentrations was partially prevented by inclusion of 240 mM NaCl, which changed the threshold concentration to ∼0.5% v/v (Fig. 1a). Time-courses showed that the reaction rates remained approximately constant for several hours after the solutions had been prepared, whether at high or low concentrations and whether in the presence or in the absence of NaCl (Fig. 1b); the yield of [^3^H]polysaccharide product only started to plateau above about 10,000 cpm, as it approached the theoretical maximum yield of product (∼17,000 cpm) from 1 kBq of [^3^H]XXXGol substrate. The slight inhibition of XET activity by NaCl, observed at high protein concentration, is unexplained. The very low activity of salt-free, low-concentration enzyme may be due to inactivation by binding to sites on the tube’s surface; we propose that above a threshold concentration essentially all these sites are already occupied by protein molecules, any additional enzyme above this threshold remains in solution and thus active.

**Fig. 2:**
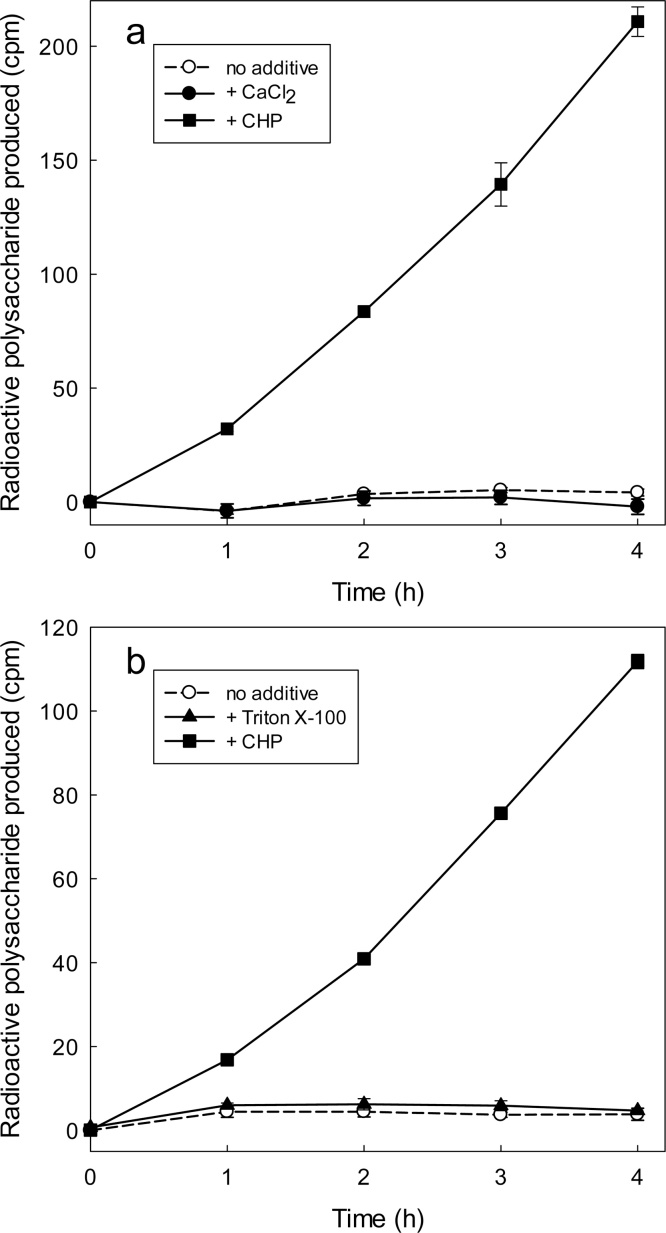
CHP, unlike CaCl_2_ and a detergent, ‘activates’ previously glass-bound XTH24 in a time-dependent way. (a) Dilute XTH24 (75 μl; protein concentration 2.16 μg/ml) was incubated in borosilicate glass tubes for 2 h to permit binding to the tube surface. The remaining free enzyme solution was then rinsed out with water and 150 μl of an XET reaction-mixture (containing xyloglucan plus [^3^H]XXXGol), either alone or supplemented with CHP (1.5 mg/ml) or 15 mM CaCl_2_, was added. At intervals, 20 μl was removed assayed for [^3^H]polysaccharide. (b) As Fig. **a** but 0.3% Triton X-100 was tested instead of CaCl_2_ [cf. critical micelle concentration of Triton X-100 ≈ 0.02%.]. In these experiments, a yield of 100 cpm of ^3^H-polysaccharide indicates 7.4 pmol XXXGol incorporated.

We confirmed that dilute AtXTH24 is indeed inactivated by binding to test-tube surfaces, where it can be re-activated by XAF-active substances. For example, in one experiment, we incubated XTH24 in a glass tube for 2 h to permit binding, then washed out any remaining soluble enzyme with water; thereafter, the ‘empty’ tube was assayed for XET activity with or without CHP, 15 mM CaCl_2_ (Fig. 2a), or 0.3% Triton X-100 (Fig. 2b). In the absence of CHP, the ‘empty’ tube exhibited no XET activity, but CHP caused a large increase in measurable XET activity; in contrast, the inorganic salt and the detergent had no effect. The rate of the XET reaction gradually accelerated during a 4-h treatment with CHP (Fig. 2a,b), rather than instantly acquiring an elevated rate as might have happened if the CHP had a direct cofactor-like or allosteric effect on the enzyme’s V_max_. This acceleration [also seen in Fig. 4, Fig. 5, which are discussed later] is compatible with a gradual release of the enzyme, by CHP, from an inactive state bound to a glass surface.

**Fig. 3:**
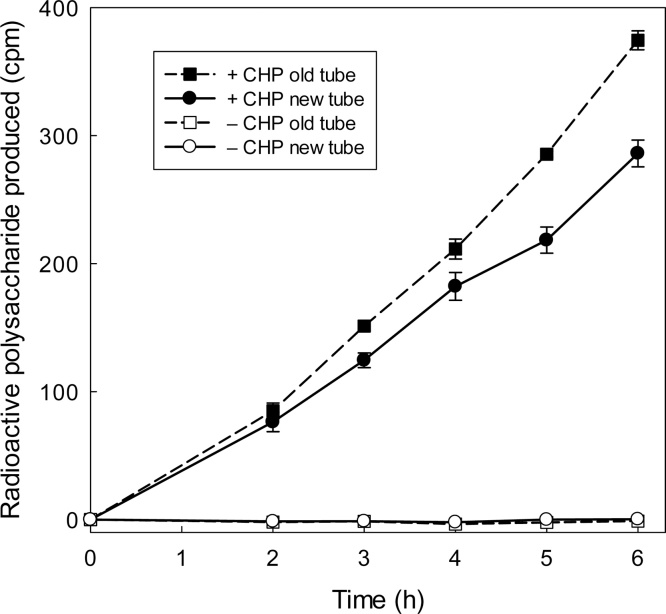
CHP releases glass-bound XTH24 into solution. A dilute solution of XTH24 (150 μl; protein concentration 2.16 μg/ml) was incubated in a borosilicate glass tube for 2 h. The tube was then rinsed with H_2_O to remove soluble enzyme, and each tube then received 300 μl of an XET reaction-mixture [100 μM [^3^H]XXXGol, 0.3% xyloglucan and 150 mM MES (Na^+^, pH 5.5); added at ‘time 0′ on the *x*-axis], either with 1.5 mg/ml CHP or without CHP. After 2 h gentle shaking at 20 °C in this (‘old’) tube, half the solution was transferred into a new tube. At intervals thereafter, samples were assayed for [^3^H]polysaccharide. A yield of 100 cpm of ^3^H-polysaccharide indicates 7.4 pmol XXXGol incorporated.

Direct evidence that CHP solubilises XTH from glass, rather than solely activating it, was obtained as follows. We incubated a dilute solution of AtXTH24 in borosilicate glass tubes for 2 h to permit binding. The enzyme solution was then discarded and the tube rinsed with water; XET assay mixture was then added in the presence or absence of CHP. After a further 2 h, to allow desorption of enzyme from the glass, half the volume was transferred into a new tube and the reaction was allowed to proceed further in both tubes. If CHP merely activated the bound XTH24 without solubilising it, the new tube would not contain any enzyme and there would be no increase in reaction products beyond the initial 2 h. If, however, CHP released the enzyme from the surface of the original tube, then reaction products would continue to be generated in both tubes beyond 2 h. The results (Fig. 3) clearly show that the reaction did indeed continue in both tubes when CHP was present, the rate in the new tube being only slightly less than that in the original tube. When CHP was omitted, there was no reaction in either tube, confirming that bound enzyme is inactive. Thus, CHP can indeed solubilise, thereby activating, AtXTH24.

**Fig. 4:**
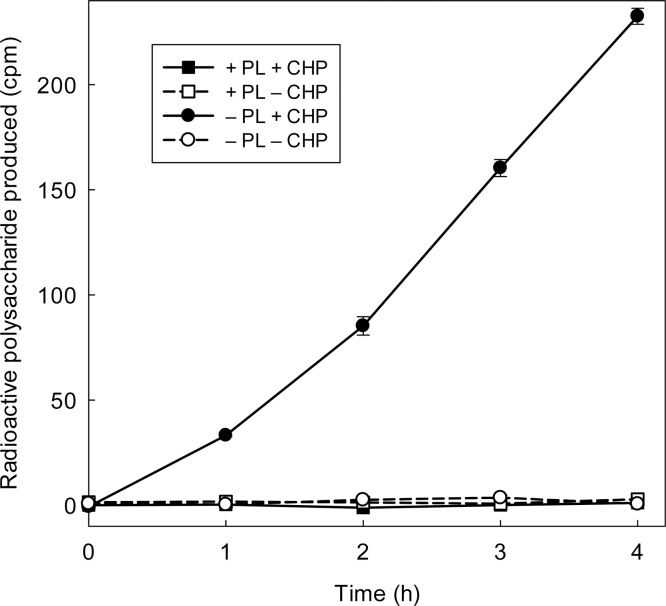
Polylysine blocks the XTH24 binding sites of glass and abolishes the CHP effect. Surface sites on borosilicate glass tubes were blocked by steeping overnight at 20 °C with polylysine (+PL; 0.5 ml, 0.05% w/v in 0.5% w/v chlorobutanol); control tubes (–PL) received 0.5 ml H_2_O. All tubes were then rinsed with 30 ml water, removing unbound PL. Dilute XTH24 (75 μl; protein concentration 2.16 μg/ml) was then incubated in each tube for 2 h, giving the enzyme the opportunity to bind to the glass, then the tubes were again rinsed with water, removing unbound enzyme. Finally, each tube received 150 μl XET assay mixture, with or without 1.5 mg/ml, and at intervals 20 μl was removed and assayed for [^3^H]polysaccharide. A yield of 100 cpm of ^3^H-polysaccharide indicates 3.7 pmol XXXGol incorporated.

**Fig. 5:**
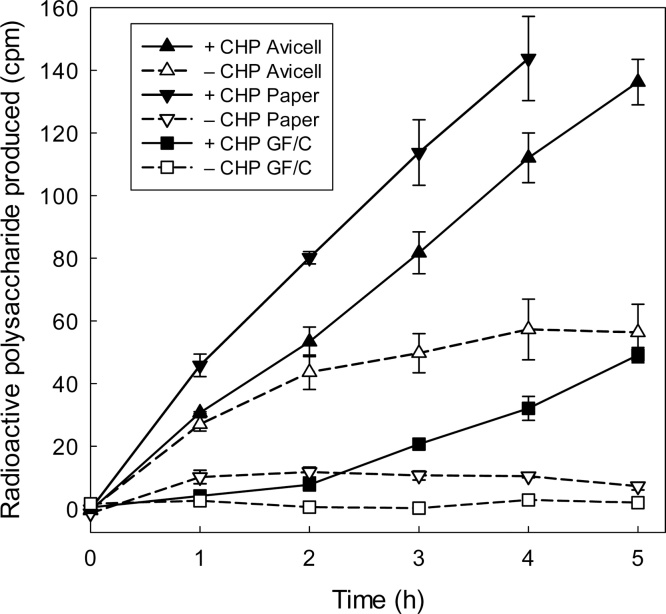
CHP ‘activates’ XTH24 previously bound to cellulose or glass-fibre. Cellulose (Avicel or Whatman No. 3 chromatography paper) or glass-fibre (Whatman GF/C) [20 mg] was incubated together with 150 μl of dilute XTH24 (protein 2.16 μg/ml) for 2 h in polylysine-blocked glass tubes. The enzyme solution was then rinsed out with a large volume of water, and 150 μl of standard XET reaction-mixture, either alone or supplemented with CHP (1.5 mg/ml), was added to the cellulose or glass-fibre. At intervals, 20 μl was removed and assayed for [^3^H]polysaccharide. In this experiment, a yield of 100 cpm of ^3^H-polysaccharide indicates 7.4 pmol XXXGol incorporated.

Polylysine (PL) was tested as an agent to block the XTH-binding sites of the glass, as it blocks extensin-binding sites (Miller and Fry, 1993). Blocking the glass surface with PL did indeed stop the AtXTH24 from binding (Fig. 4). Furthermore, CHP had no effect on the XET activity of a dilute AtXTH24 solution in PL-blocked glass tubes (Fig. 4), supporting the idea that the action of CHP was due to its ability to prevent surface binding. We interpret the data as follows: in both the +PL tubes, all XTH had been washed out by the water rinse, so no XET activity was possible; in the −PL tube without CHP, glass-bound XTH was present but it remained bound and therefore inactive; and only in the −PL +CHP tube was there any bound enzyme to be brought into solution (thus re-activated) by the CHP.

It could *a priori* be argued that PL might itself affect XET activity, irrespective of its effect on glass binding, accounting for the above results. However, this hypothesis was disproved in experiments in which the unbound enzyme was not washed out of the tubes (Fig. S1), and in which supplementary PL was present in soluble form (Fig. S2).

Besides obtaining evidence for binding to vial surfaces (Fig. 1, Fig. 2, Fig. 3, Fig. 4 and S2), we also showed that AtXTH24 can be inactivated by binding to glass-fibre and cellulose — from which it was re-activated by CHP (Fig. 5). The XET activity restored by CHP in this experiment may even have been underestimated if some of the donor substrate xyloglucan became adsorbed to the cellulose. The loss of activity by binding to cellulose is particularly interesting as this could mimic the behaviour of XTHs in the cell wall *in vivo*, and represent a biological role for the active component of CHP.

### 3.2 Plant cell walls contain bound, inactive XTHs which CHP can reactivate by solubilisation

**Fig. 6:**
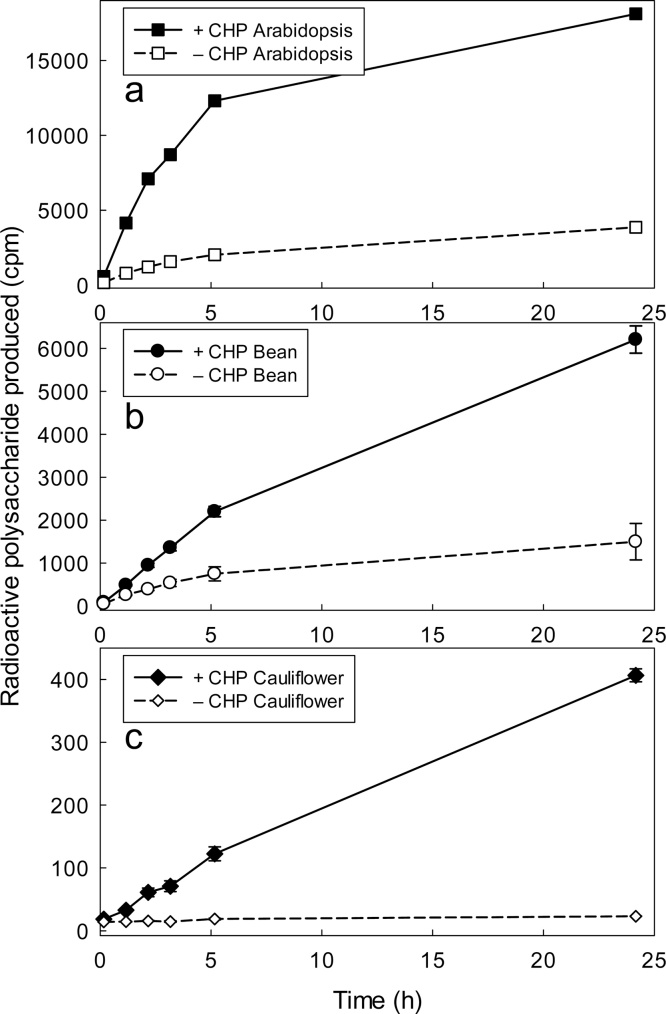
CHP activates wall-bound XTHs present in diverse plant tissues. Tissue from (a) 7-day suspension-cultured *Arabidopsis* cells, (b) mung bean shoots and (c) cauliflower florets was homogenised, frozen, thawed and copiously water-washed. The crude wall preparations, in PL-blocked PCR tubes, were gently shaken with standard XET reaction-mixture with or without CHP (1.5 mg/ml). At intervals the yield of soluble [^3^H]polysaccharide product was assayed. In this experiment, a yield of 100 cpm of ^3^H-polysaccharide indicates 1.2 pmol XXXGol incorporated.

**Fig. 7:**
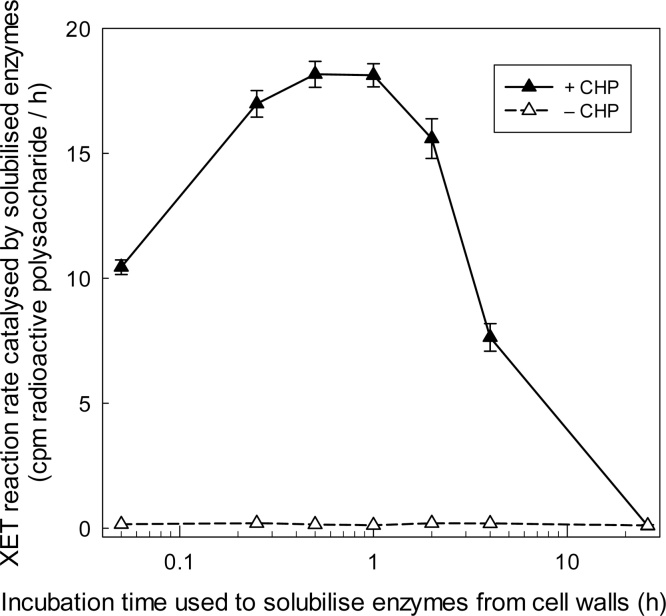
CHP transiently solubilises XTHs from a crude cell-wall preparation of suspension-cultured *Arabidopsis* cells. Tissue from 3-day *Arabidopsis* cell suspension-cultures was frozen, thawed, copiously water-washed, dispensed into PL-blocked 1.5-ml polypropylene tubes, and gently shaken in buffer [150 mM MES (Na^+^, pH 5.5) containing 100 μM non-radioactive XXXGol and 0.3% tamarind xyloglucan] with and without CHP (1.5 mg/ml). At intervals (shown on *x*-axis), 70 μl of supernatant was transferred into a PL-blocked PCR tube, frozen until all samples were ready, then thawed; 1 kBq [^3^H]XXXGol was added and incubation was continued at 20 °C. At 0, 2, 4 and 24 h, a 15-μl sample was assayed for [^3^H]polysaccharide product. The reaction in each case was approximately linear, and the gradient (linear regression ± its SE; df = 2) was calculated (plotted on *y*-axis). In this experiment, a yield of 100 cpm of ^3^H-polysaccharide indicates 2.3 pmol XXXGol incorporated.

An ability of XTHs to bind to biologically relevant surfaces in the plant cell wall, suggested by the above observations on cellulose, was confirmed by direct observation. Crude cell-wall preparations from a range of plant materials (cauliflower florets, mung bean shoots and 7-day-old *Arabidopsis* cell-suspension cultures) were freed of soluble XTHs by homogenising, freezing, thawing and copious water-washing. When the washed, cell-wall-rich residue was incubated in standard XET reaction mixture, only low XET activity was detected, confirming the effectiveness of the washing procedure and the inability of any remaining insoluble XTH to act on exogenous substrates. However, the measureable XET activity was strongly increased by exogenous CHP (Fig. 6). We suspected that this enzyme activation was due to solubilisation, as in the case of glass-bound XTH24; and this was confirmed for 3-day-old *Arabidopsis* cultures, where the CHP-‘activated’ XET was detected in the soluble supernatant after centrifugation (Fig. 7).

CHP was able to solubilise XET activity from washed *Arabidopsis* cells very rapidly, but the soluble activity was subsequently lost if the solution was left in contact with the crude cell walls for more than about 2–4 h (Fig. 7). Similar results were obtained for cultured *Arabidopsis* cells in various stages of their growth cycle (2, 3, 7 and 9 days after sub-culturing; Fig. 6, Fig. 7 and data not shown). We propose that the CHP was able to solubilise XTHs from its natural binding sites in the cell walls (such as the cellulose–xyloglucan network), but was unable to prevent the solubilised enzymes re-binding to a different site in the crude cell-wall preparation (*e.g. via* ionic bonds to nuclei, which XTHs would have access to only after the cells had been homogenised).

### 3.3 XAF activity of CHP in comparison to NaCl

**Fig. 8:**
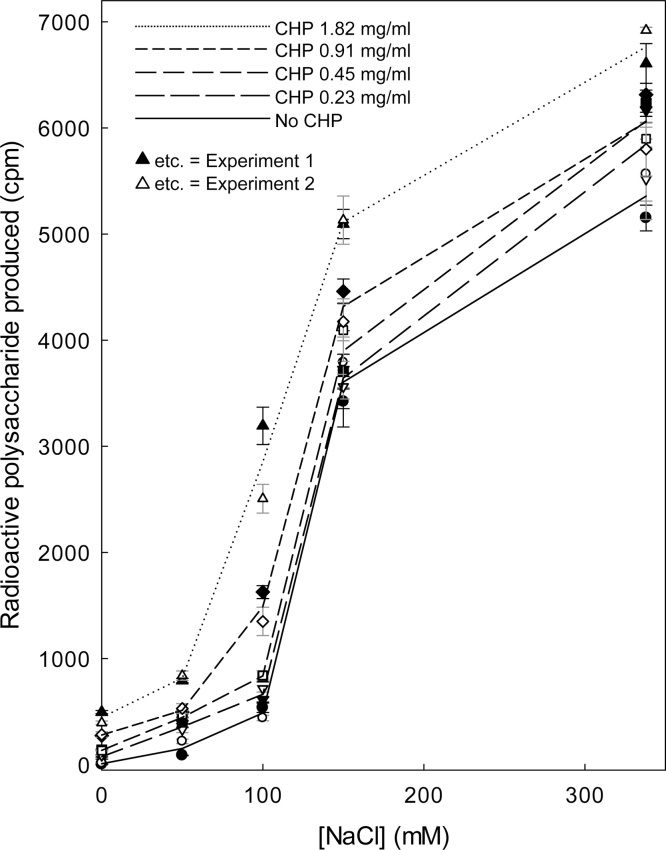
CHP and NaCl synergistically solubilise XET activity from *Arabidopsi*s cell walls. Frozen/washed *Arabidopsis* cells were incubated for 1 h in 66 μl of buffer [183 mM MES (Na^+^), pH 5.5] containing various combinations of CHP and NaCl. After centrifugation, 20 μl of supernatant was incubated with XET reaction mixture and the yield of [^3^H]polysaccharide at 16 h was determined ±SE. The experiment was carried out five times with similar results; data from two runs (‘experiments 1 and 2′, represented by filled and open symbols respectively) are combined here. The lines interconnect the means of the two experiments. In these experiments, the volume sampled had contained 1.0 kBq [^3^H]XXXGol (carrier-free); the theoretical maximum yield of polysaccharide product was about 17,000 cpm.

The ability of CHP to solubilise XTHs from *Arabidopsis* cell walls was mimicked by an inorganic salt, NaCl (Fig. 8), as reported earlier for dilute solutions of desalted enzyme (Takeda and Fry, 2004). The effect of NaCl plateaued above about 150–200 mM, but the CHP effect did not plateau even at the highest concentration tested (1.8 mg/ml, whose conductivity indicated an ionic strength equivalent to 9 mM NaCl). In the absence of CHP, 100 mM NaCl was about as effective as 1.8 mg/ml CHP (≈9 mM ionic strength) in the absence of NaCl. Thus, the CHP was certainly not acting through a simple ionic mechanism. The fold-effect of CHP was greatest in the absence of NaCl, but strong CHP effects (4.6- to 9.1-fold promotion by 1.8 mg/ml CHP) — and much higher absolute XET activities — were still detected in the presence of 50–100 mM NaCl, indicating synergy between CHP and the inorganic salts (Table S1). This observation, together with the previous finding that certain anionic polysaccharides promote the XET activity of de-salted XTH preparations particularly well if a sub-optimal concentration of NaCl is also present (Takeda and Fry, 2004), suggested that XAF activity should be routinely assayed by suspending the washed *Arabidopsis* walls in a solution containing 75 mM NaCl [buffered with 200 mM MES (Na^+^), pH 5.5, which itself has a low ionic strength and has no appreciable XAF activity (Takeda and Fry, 2004; and our unpublished work)]. The data show that CHP can solubilise XTHs from washed *Arabidopsis* walls, independently of any simple ionic effect, and that solubilisation causes these enzymes to acquire detectable XET enzymic activity.

### 3.4 XAF activity of CHPs from diverse plants

To test whether XAF activity is widespread across the plant kingdom, we prepared CHPs from a selection of organs, and cell-suspension cultures, from various plant species (Table 1). The yield of cold-water-extractable, heat-stable polymer varied over a 10-fold range (0.24–2.4 mg CHP per g fresh weight). All CHPs obtained exhibited XAF activity, successfully solubilising *Arabidopsis* XTHs: when each CHP was tested at 2.0 mg/ml, a 6-fold range of XAF activities was obtained, resulting in readings of 500–3000 cpm [^3^H]polysaccharide in the XET assays (cauliflower, our standard CHP source, gave 872 cpm). Thus, XAF appears to be ubiquitous in the plant kingdom.

The XAF activity of *Arabidopsis* CHPs, assayed at 2 mg/ml, varied approximately 3-fold between organs, the order of effectiveness being: leaves > stems > flowers > cell-cultures (Table 1). This indicates some developmental control of XAF. On the other hand there was relatively little difference in efficacy between the CHPs from very young and mature celery leaves.

Different CHP preparations (always tested at 2.0 mg/ml in de-ionised water) differed in conductivity over a ∼10-fold range (equivalent to 1.4–15 mM NaCl). It is clear from Table 1 that the XAF activity does not closely correlate with measured conductivity of the CHP solution. Moreover, the CHPs at 2 mg/ml had conductivities considerably lower than that of the MES buffer in which they were assayed for XAF activity, confirming that the XAF phenomenon is not a simple ionic effect but is due to specific chemical features of the CHPs.

## 4 Discussion

### 4.1 XTH activating factor (XAF) solubilises bound XTH *in vitro*

Takeda and Fry (2004) reported that the XET activity of XTH solutions diminished during de-salting, and that the lost activity could be largely restored by low-M_r_ salts, certain acidic polysaccharides and CHP (a cold-water-extractable polymer preparation from cauliflower florets). It was not determined whether the restorative effect of these agents was due to a promotion of the activity of soluble enzyme or to a re-solubilisation of sequestered enzymes. We now report that dilute solutions of XTHs temporarily lose their activity by binding to any of a range of surfaces, including glass and cellulose, and that the activity can be restored if the enzymes are re-solubilised from these surfaces by salts and/or polymers. We introduce the term XAF (XTH activating factor) for substances with this capability.

Binding to borosilicate glass is found to be minimised by pre-treatment of the glass with polylysine, suggesting that this artificial polypeptide can occupy the sites that would otherwise sequester XTHs. However, the nature of the protein–glass bonding is not known.

Although some of the polymers in CHP are acidic, we show that the XAF effect of their solution is far greater than predicted by the ionic strength and is thus not a simple salt effect. Furthermore, CHP and NaCl synergise in their XAF effects, indicating that they act *via* different mechanisms.

The binding of XTHs to solid surfaces is not simply an experimental artefact that results in enzyme losses during purification attempts. A biological relevance was first suggested by the discovery that XTHs can temporarily lose activity by binding to cellulose (both Avicel and filter paper), a major component of the cell walls that contain the xyloglucan chains on which XTHs act *in vivo*. We show that CHP exerts its XAF effect on cellulose-bound XTHs *in vitro*, and we suggest that it may also do so *in vivo*.

More direct evidence for a biologically relevant sequestration of XTHs in the plant cell wall was provided by a study of crude cell-wall preparations, thoroughly washed free of soluble XTHs. All three cell-wall sources tested (mung bean shoots, cauliflower florets and suspension-cultured *Arabidopsis* cells) release XET activity into solution on treatment with CHP. Wall-bound XTHs show much lower activity on exogenous substrates in the absence of an XAF source.

CHP solubilises the XTHs from *Arabidopsis* culture cell walls rapidly, the yield peaking at about 0.5 h. Thereafter, the solubilised enzyme loses activity and has become completely inactive by 24 h. We have repeatedly shown that XTHs are not denatured over this time period, so long as they remain soluble. We therefore propose that the XAF activity of CHP is able to solubilise the enzyme from natural binding sites in the cell wall, but that the enzyme subsequently binds elsewhere, through bonds that cannot be disrupted by CHP. These newly formed bonds are unlikely to be to the surface of the vials used, since these had been surface-blocked with polylysine, a treatment which prevents an XTH solution (freed of cell walls) losing XET activity even during 24-h incubations. Instead, we propose that the solubilised XTHs can artefactually bond to different sites in the crude ‘cell wall’ preparation, such as *via* ionic bonds to contaminating nuclei, to which wall-localised XTHs do not have access *in vivo*. These ionic bonds would not be broken by CHP, which at the concentrations used had an ionic strength of less than 10 mM. The synergy noted between CHP and NaCl (Fig. 8) also argues in favour of these two agents breaking (or preventing the formation of) different types of bond between the XTHs and the crude cell-wall preparations.

### 4.2 Novel methodology

A convenient and biologically pertinent novel assay for XAF activity was developed. In this method, we froze and thawed *Arabidopsis* cells to disrupt the plasma membrane and then all soluble materials were washed away with water, leaving a preparation containing cell walls with their firmly bound proteins as well as other water-insoluble components such as starch grains, membranes and chromatin. When the wall-enriched *Arabidopsis* preparation was incubated with salts or CHP containing XAF, XTH proteins were rapidly solubilised and the supernatant was assayed for soluble XET activity by a standard radiochemical procedure (Fry et al., 1992). In the absence of salts or CHP, almost no buffer-soluble XET activity was detected, proving that the *Arabidopsis* cell walls had been successfully washed free of unbound enzymes. Thus, any buffer-soluble XET activity observed after the incubation of the walls with XAF was due to the solubilisation of wall XTHs. The method was facilitated by the use of 96-well plates allowing us to deal with a large number of samples in small volumes (66 μl) simultaneously. Replication was good, and error bars were small. With this method, we are able to test the solubilisation of *Arabidopsis* wall-bound XTHs *in vitro* by any putative XAF, salts etc. This method can now be used to further characterise endogenous XAF.

### 4.3 Biological significance of XAF *in vivo*

In the light of the *in-vitro* experiments, we propose that biologically relevant bonding of XTHs occurs within the cell wall, that this bonding is not simply ionic but can be disrupted by CHP, and that XTHs sequestered in this way are unable to exhibit high XET activity. Indeed, Farkaš’s group showed that xyloglucan–XTH complexes can be purified by transient binding to the cellulose of paper towels (Sulová and Farkaš, 1999). We suggest that XAF-active polymer(s) present in CHP can solubilise, and thereby activate, wall-bound XTHs. It is understandable that XTHs which are immobilised within the plant cell wall, especially if attached to the cellulosic microfibrils, exhibit little or no XET activity since they are unable to access more than a single near-neighbour xyloglucan chain. By rendering the enzyme mobile, XAF can permit the enzyme to diffuse through the wall matrix until it encounters an accessible xyloglucan chain to use as donor substrate, with which it then forms a xyloglucan–enzyme covalent complex (Sulová et al., 1998). When this complex (now the reducing end of a xyloglucan chain) comes within reach of the non-reducing end of another xyloglucan chain that has an appropriate chemical structure to serve as acceptor substrate, an inter-polymeric transglycosylation can be completed. This model envisages XAF playing a pivotal role in allowing XTH to achieve inter-polymeric transglycosylation — a process demonstrated to occur *in vivo* (Thompson and Fry, 2001) but difficult to envisage with a cellulose-bound enzyme in the absence of XAF.

## 5 Conclusions

We conclude that XTHs have a strong propensity to bind *in vitro* to any of numerous surfaces, including glass, polypropylene and cellulose, thereby losing enzymic activity. In addition, plant cell walls natively contain bound, inactive XTHs. Sequestered XTHs in both these situations are re-solubilised into active form by treatment with XAF and/or high concentrations of NaCl. XAF is a cold-water-extractable plant polymer that cannot be inactivated or coagulated by boiling and is thus not a typical protein. Its ready extractability in cold water, *e.g.* from cauliflower florets, indicates that it is not a structural component of the plant cell wall, though it may be present as an apoplastic solute such as an arabinogalactan-protein. We propose that XAF may play a role *in vivo* by re-activating XTHs that had been taken out of play by wall-binding, and thus serve as a biologically important regulator of plant cell-wall assembly, restructuring and expansion. We have developed a simple, relatively high-throughput, quantitative assay for XAF activity, which will aid further characterisation of the to-date unidentified active principle of XAF.

## Authors’ contribution

SCF conceived and designed the research and wrote the manuscript. All authors contributed to performing the experiments and commented on the manuscript.
