# Supplementary material for: Xyloglucan endotransglucosylase/hydrolases (XTHs) are inactivated by binding to glass and cellulosic surfaces, and released in active form by a heat-stable polymer from cauliflower florets
Source: J Plant Physiol. 2017 Nov;218:135–43. doi: 10.1016/j.jplph.2017.07.022 (PMC5669584; doi:10.1016/j.jplph.2017.07.022)
Supplement: Supplementary file 1 [file mmc1.pptx]

## Slide 1
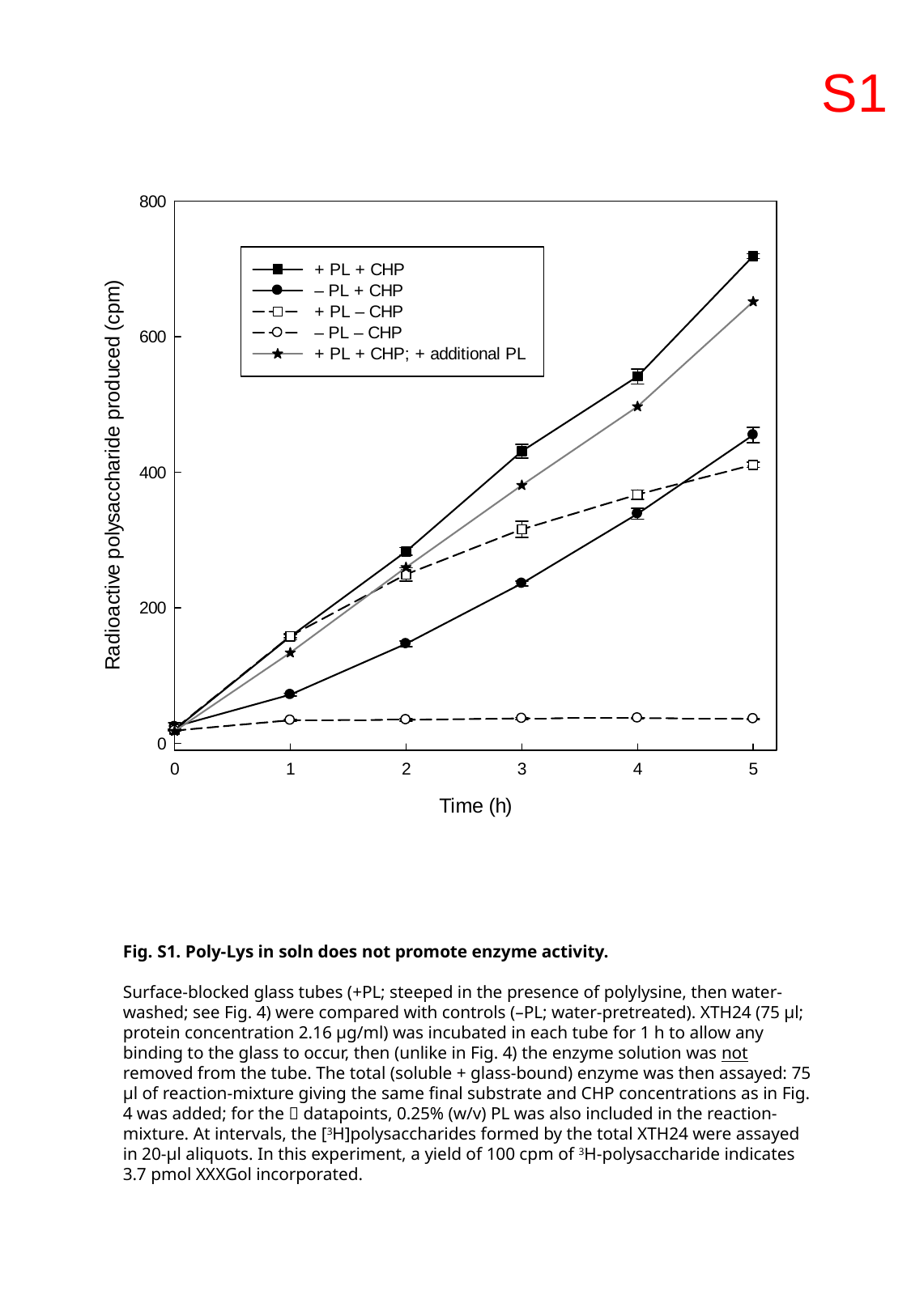

S1
Fig. S1. Poly-Lys in soln does not promote enzyme activity.
Surface-blocked glass tubes (+PL; steeped in the presence of polylysine, then water-washed; see Fig. 4) were compared with controls (–PL; water-pretreated). XTH24 (75 µl; protein concentration 2.16 µg/ml) was incubated in each tube for 1 h to allow any binding to the glass to occur, then (unlike in Fig. 4) the enzyme solution was not removed from the tube. The total (soluble + glass-bound) enzyme was then assayed: 75 µl of reaction-mixture giving the same final substrate and CHP concentrations as in Fig. 4 was added; for the  datapoints, 0.25% (w/v) PL was also included in the reaction-mixture. At intervals, the [3H]polysaccharides formed by the total XTH24 were assayed in 20-µl aliquots. In this experiment, a yield of 100 cpm of 3H-polysaccharide indicates 3.7 pmol XXXGol incorporated.

## Slide 2
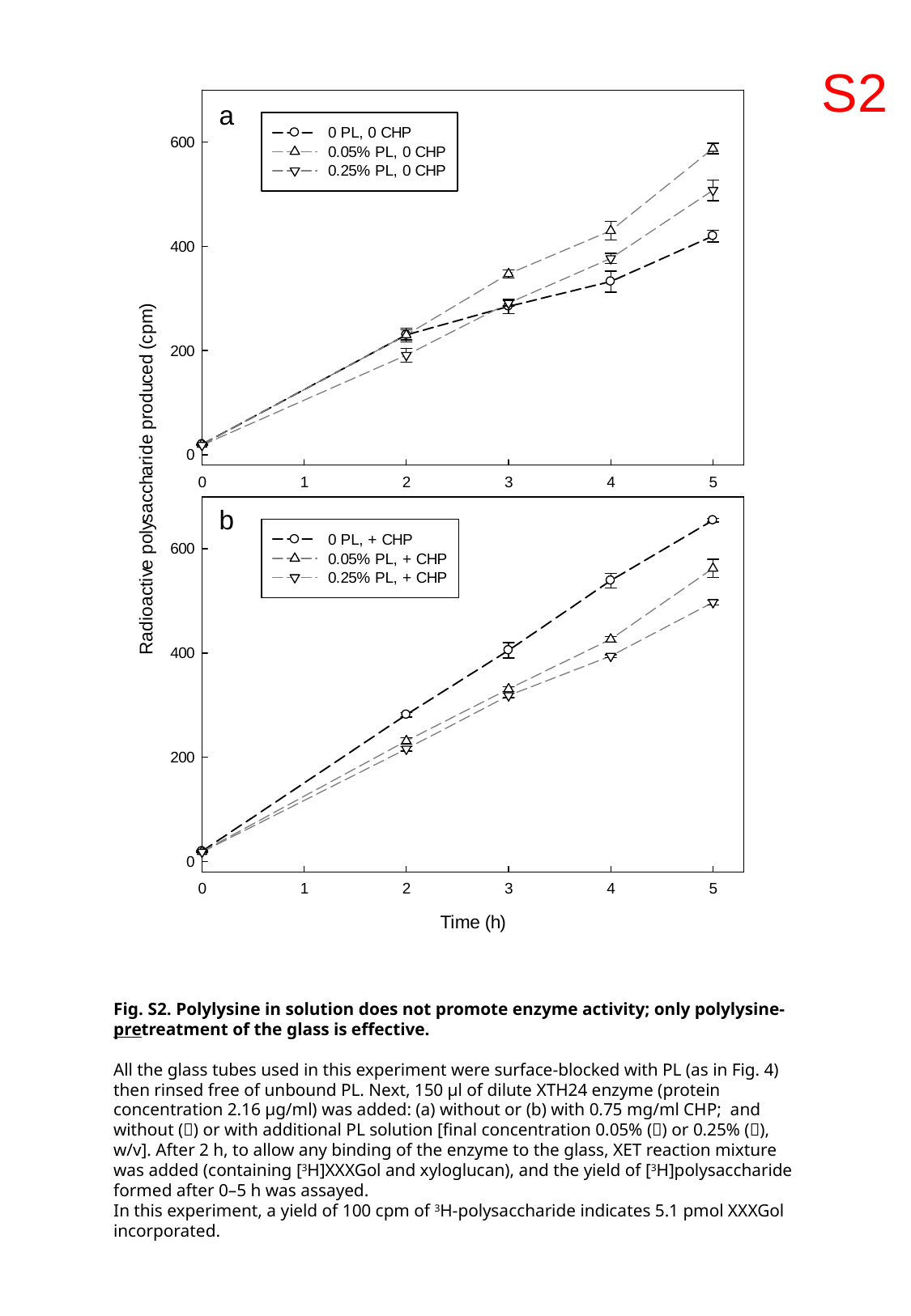

S2
a
b
Fig. S2. Polylysine in solution does not promote enzyme activity; only polylysine-pretreatment of the glass is effective.
All the glass tubes used in this experiment were surface-blocked with PL (as in Fig. 4) then rinsed free of unbound PL. Next, 150 µl of dilute XTH24 enzyme (protein concentration 2.16 µg/ml) was added: (a) without or (b) with 0.75 mg/ml CHP; and without () or with additional PL solution [final concentration 0.05% (ρ) or 0.25% (), w/v]. After 2 h, to allow any binding of the enzyme to the glass, XET reaction mixture was added (containing [3H]XXXGol and xyloglucan), and the yield of [3H]polysaccharide formed after 0–5 h was assayed.
In this experiment, a yield of 100 cpm of 3H-polysaccharide indicates 5.1 pmol XXXGol incorporated.
